# Versatility of the Cyano Group in Intermolecular Interactions

**DOI:** 10.3390/molecules25194495

**Published:** 2020-09-30

**Authors:** Steve Scheiner

**Affiliations:** Department of Chemistry and Biochemistry, Utah State University, Logan, UT 84322-0300, USA; steve.scheiner@usu.edu; Tel.: +1-435-797-7419

**Keywords:** tetrel bond, molecular electrostatic potential, π-hole, NMR chemical shift, charge transfer, SAPT

## Abstract

Several cyano groups are added to an alkane, alkene, and alkyne group so as to construct a Lewis acid molecule with a positive region of electrostatic potential in the area adjoining these substituents. Although each individual cyano group produces only a weak π-hole, when two or more such groups are properly situated, they can pool their π-holes into one much more intense positive region that is located midway between them. A NH_3_ base is attracted to this site, where it forms a strong noncovalent bond to the Lewis acid, amounting to as much as 13.6 kcal/mol. The precise nature of the bonding varies a bit from one complex to the next but typically contains a tetrel bond to the C atoms of the cyano groups or the C atoms of the linkage connecting the C≡N substituents. The placement of the cyano groups on a cyclic system like cyclopropane or cyclobutane has a mild weakening effect upon the binding. Although F is comparable to C≡N in terms of electron-withdrawing power, the replacement of cyano by F substituents substantially weakens the binding with NH_3_.

## 1. Introduction

Following many decades of study of the H-bond [1,2,3,4,5,6,7], a great deal of research has turned to closely related interactions in which the bridging proton is replaced by any of a number of larger atoms, generally drawn from the right side of the periodic table. Depending upon the specific column of the table in which these atoms lie, the noncovalent bonds are typically referred to as halogen, chalcogen, and pnicogen bonds [8,9,10,11,12,13,14,15,16,17,18]. Despite the diversity of the bridging atoms, these various interactions share a number of characteristics. Using the halogen bond as an example, when bound to a substituent R, the halogen atom X is characterized by a highly anisotropic charge distribution. While the X atom is surrounded by a negative region of electrostatic potential on its equator, a positive region occurs along the extension of the R-X bond. This positive polar section is commonly termed a σ-hole [19,20] and is one of the most important aspects that allows a nucleophile to approach the X atom and engage in a halogen bond. This same phenomenon occurs on a divalent chalcogen Y atom, but each of its two RY bonds leads to a separate σ-hole, and a logical extension provides three σ-holes around a Z pnicogen atom in its ZR_3_ configuration. Recent works [21,22,23,24,25,26,27,28,29,30] have also included tetrel atoms T (C and Si, etc.) in this category, as the tetravalent TR_4_ molecule has four σ-holes, so could, at least in principle, participate in as many as four simultaneous tetrel bonds. One of the chief features of these σ-holes is that they are highly subject to the nature of the R group that lies directly opposite them and that produces them. A highly electron-withdrawing group intensifies the hole, while the opposite is true for an electron-releasing substituent.

While σ-holes serve as a simple way to understand the attraction toward a nucleophile, it is by no means the only vehicle toward a noncovalent bond. The same electrostatic attraction would apply to any positive region of the surrounding electrostatic potential, regardless of its origin. One of the more common alternative types of positive areas concerns planar systems. A typical example would be H_2_CO or H_2_CS. The potential of each contains a positive area [31] lying above (and below) the plane of the molecule, close to the C atom. Due to its position, this region is referred to as a π-hole. The same sort of tetrel bonding through a C π-hole occurs in other systems as well [32,33]. This idea is not restricted to C but occurs on other sorts of atoms—for example, the S atom of SO_2_ [31] or SO_3_ [34] or the central O of ozone [35] or for tetrel T atoms larger than C, as in F_2_TO [36,37,38], or the N atom of the -NO_2_ group [39]. Other planar examples involve triel (Tr) atoms in TrR_3_, where R indicates some substituent [40,41]. 

Nor is there a limitation to planar geometries, as π-holes are also common in linear molecules, such as the area above the C atom of CO_2_ [42,43,44,45,46,47,48], OCS [49,50,51], N_2_O, and CS_2_ [52,53,54]. Just as their σ-hole analogs facilitate attractions toward a nucleophile, the same is true of these π-holes.

Within the framework of linear systems, the cyano group has some particularly intriguing characteristics. On one hand, it is very strongly electron-withdrawing, so its placement on a molecule would tend to produce an intense σ-hole opposite to it [55]. Its N terminus contains a negative region along the extension of the C≡N axis, which can act as an electron donor [16,17,36,55,56,57,58,59]. The C atom of a C≡N-Ph can even serve as an electron donor in a halogen bond with I [60]. The π-system of the C≡N bond is able to engage in π-hole interactions with a nucleophile [61,62].

It is the latter characteristic that is perhaps the most intriguing. The positive region lying off of the C≡N axis does not lie midway between the C and N atoms. Rather, it is situated away from the N atom, closer, in fact, to the R substituent in the RC≡N molecule [62]. Moreover, the positive area is not intense at all, generally unable to hold a nucleophile. For example, when a NH_3_ base is placed in this region, it moves away, so as to engage in a NH··N H-bond with the terminal N atom, which is the mainstay of the complex.

Given these observations, a question that arises is whether two or more cyano groups, when positioned appropriately, can reinforce one another’s positive regions to form a joint π-hole that is stronger than either individually. This issue was a theme in some recent work by the Mooibroek and Frontera groups [23,63,64,65,66], who placed a number of cyano groups on adjacent C atoms of a cyclopropyl system. The authors found that a nucleophile then bound fairly strongly to a site between these cyano groups. However, their analysis led them to conclude that the binding occurred not with the C≡N but, rather, with the C-C bond of the cyclopropane ring.

These general ideas and the aforementioned specific work raise some interesting questions. Is there something unique to the intense strain within the cyclopropane system that is an important element in this binding? If the binding of the nucleophile chiefly involves the cyclopropyl C-C bond, can a F substituent, also strongly electron-withdrawing, serve the same function as C≡N? It would be interesting to determine whether the multiplicity of the C-C bond is a factor; will this bonding occur for C=C or C≡C bonds as well? As the previous work focused on a nucleophile occupying a site between four C≡N groups, is this the minimum required, or can the same sort of binding occur with fewer such substituents? What would be the bare minimum?

These questions are addressed through quantum chemical calculations of model systems. Beginning with simple alkanes, various numbers of cyano substituents are added to one or both C atoms of ethane. Similar additions were made to the double- and triple-bonds of ethylene and acetylene. So as to consider the effects of ring systems of various sizes, cyano groups were also added to cyclopropane and cyclobutane. Adding another dimension, C≡N groups were replaced by F, so as to distinguish the differing effects of these two electron-withdrawing substituents. For purposes of uniformity, NH_3_ was chosen as the universal base that is allowed to interact with these Lewis acids. NH_3_ is small enough so as to avoid complicating secondary interactions, yet is a strong enough nucleophile to bring out the binding properties of the various acids. 

## 2. Results

The calculations described below are organized as follows. The NH_3_ base is first permitted to interact with a substituted ethane in which all six H atoms were replaced by C≡N. Then, two of the cyano groups are removed, leaving four substituents, either C≡N or F. Other molecules considered next contain three or less substituents on the alkane. The succeeding section evaluates the effect of changing the single C-C bond of the alkane to C=C and C≡C. The next section considers how the binding might change if the carbon framework is made cyclical, as in cyclopropane and cyclobutane. The last section describes how the formation of the various interactions affect the internal properties of the monomers.

### 2.1. Hexa-Substituted

**Figure 1:**
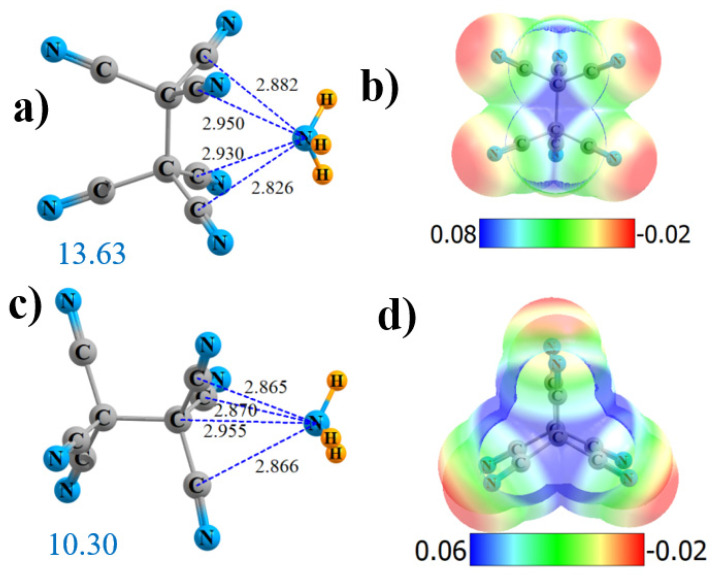
Geometries and monomer molecular electrostatic potential (MEP) of (CN)_3_C-C(CN)_3_ interacting with NH_3_ from the side (**a**,**b**) and end (**c**,**d**). Dotted lines denote AIM bond paths. Large blue numbers indicate interaction energy in kcal/mol. Distances in Å and MEP in au. MEP can be converted to kcal/mol by factor of 627.51.

There are two locations to which NH_3_ can bind to the hexa-substituted (CN)_3_C-C(CN)_3_. The preferred site is “side-on” between the two C(CN)_3_ groups, where it can interact directly with four C atoms. As shown in Figure 1a, the N is located nearly symmetrically, roughly 2.9 Å from all four. The molecular electrostatic potential (MEP) of this molecule presented in Figure 1b indicates a strong symmetrically located maximum (in blue) with a V_max_ of 60.7 kcal/mol. There is another maximum on the MEP that lies along the C-C axis extension, where the NH_3_ is symmetrically disposed around the three C≡N groups of one end of the molecule. As seen in Figure 1c, the N lies about 2.87 Å from the C atoms of the three C≡N groups and 2.955 Å from the terminal C in this end-on geometry. This site echoes the MEP, which contains another maximum along the C-C bond extension, with V_max_ = 46.9 kcal/mol. Fully in keeping with the intensities of the two MEP maxima, the structure in Figure 1a is bound more tightly than that in Figure 1b by 3.3 kcal/mol, as indicated by the large blue numbers in Figure 1a,c. The interaction energies and values of V_max_ are displayed explicitly in Table 1, following the number n of C≡N groups with which the NH_3_ interacts. It should be emphasized that, in Figure 1 and in those that follow, dotted lines denote the presence of an AIM bond path.

The nature of the bonding may be understood in a number of ways. First, on the basis of AIM analysis, Figure S1a displays bond paths between the N of NH_3_ and each of the four of the C atoms of the C≡N groups (denoted C_N_), which are in its vicinity. The fourth column of Table 1 indicates that the value of the density at each of these four bond critical points is 0.013 au. The NH_3_ also forms four bond paths in the end-on configuration. As illustrated in Figure S1b, three of these are to C≡N groups (ρ_BCP_ = 0.0135 au) and the fourth to alkane C, with a density of 0.0131, very similar to the four values for the side-on structure. 

Another perspective on the binding comes from a natural bond orbital (NBO) analysis. Second-order perturbation values of E(2) for transfers from the NH_3_ N lone pair are provided in the penultimate column of Table 1. The picture is consistent with the AIM analysis in that transfer occurs into the π*(CN) antibonding orbitals of each of the neighboring CN groups: four for the side-on and three for end-on. Bonding in the side-on geometry is supplemented by transferring into the C-C_N_ antibonding orbitals connecting two of the cyano groups to alkane C. The AIM bond path to alkane C in the end-on structure is reflected in the transfer into the pertinent σ*(CC) antibonding orbital. The overlap of the N lone pair with one of these π*(C≡N) orbitals is pictured in Figure S2a, and an analogous diagram in Figure S2b depicts the overlap with the σ*(C-C) antibond that occurs in the end-on structure. Therefore, in both geometries, the bulk of the direct bonding occurs to the C≡N groups. There is a small supplement to the alkane C atom in the end-on geometry, but this represents only a small fraction. In either case, the bond might be categorized as a tetrel bond, since it is a C atom that serves as a primary electron acceptor. The last column of Table 1 contains the total charge transferred from the NH_3_ nucleophile to the Lewis acid. This charge amounts to just under 0.05 e, with a slight edge toward the side-on geometry.

### 2.2. Tetrasubstituted

**Figure 2:**
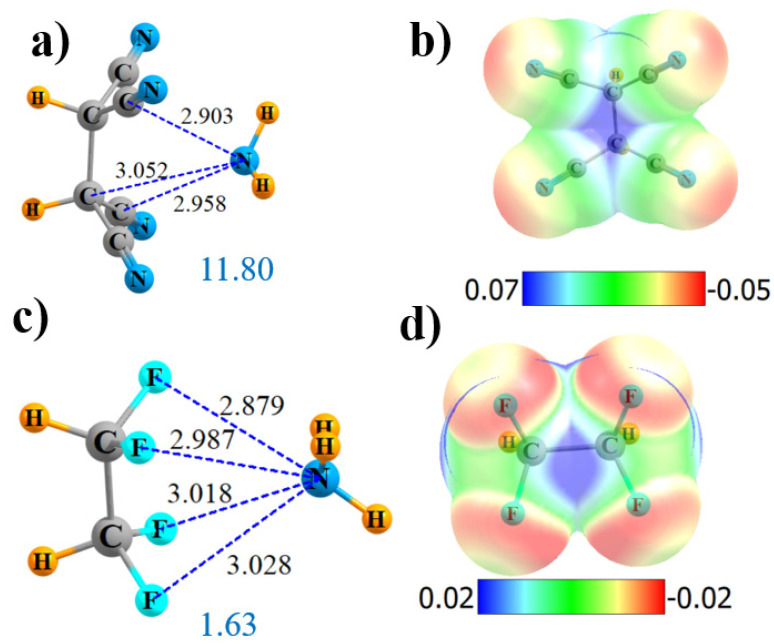
Geometries and monomer MEP of complex of NH_3_ with (CN)_2_HC-CH(CN)_2_ (**a**,**b**) and F_2_HC-CHF_2_ (**c**,**d**).

There is some question as to the value in having six C≡N substituents, since there are, at most, four that can interact directly with the NH_3_. If the two CN groups are removed from the side-on geometry in Figure 1a, the structure illustrated in Figure 2a is obtained. The location of the NH_3_ is consistent with the position of the positive blue area of the MEP of (CN)_2_HC-CH(CN)_2_ in Figure 2b. AIM removed two of the four N··C_N_ bond paths in Figure S1c, adding one to one of the nearby alkane C atoms. NBO paints a slightly different picture. In the first place, all four of the N_lp_→π*(C≡N) transfers remain. NBO also adds two more transfers into the σ*(CH) antibonding orbitals, which are portrayed in Figure S2c. However, nonetheless, the interaction with (CN)_2_HC-CH(CN)_2_ is weakened relative to the side-on (CN)_3_C-C(CN)_3_ by 1.8 kcal/mol, which can be attributed, in part, to the reduced V_max_ of 44.7 kcal/mol as compared to 60.7 kcal/mol. A symmetry-adapted perturbation theory (SAPT) partitioning confirms the reduction of the electrostatic component of the interaction, dropping from 22.4 kcal/mol for (CN)_3_C-C(CN)_3_ down to 18.6 kcal/mol for (CN)_2_HC-CH(CN)_2_. The reduction in overall interaction energy is also reflected in a decrease in the total amount of charge transferred, down to 0.035 e. 

Like C≡N, F is also highly electron-withdrawing, so the four cyano groups of (CN)_2_HC-CH(CN)_2_ were replaced by F atoms, leading to the structure in Figure 2c. The exchange of the CN groups by F severely reduces the V_max_ down to only 17.7 kcal/mol, while leaving it midway between the two alkane C atoms (see Figure 2d). Both AIM (Figure S1d) and NBO agree that there are four N··F noncovalent bonds present, although considerably weaker than those involving CN groups. The bond critical point densities are below 0.01 and the NBO E(2) less than 0.3 kcal/mol. Note, however, that NBO adds two other small increments, which involve transfer from the N lone pair to the CH antibonding orbitals of the H atoms that are turned away from NH_3_. NBO also suggests that there may be a weak NH··F H-bond that contributes to the stability. However, F is a poor substitute for the CN group in terms of energetics, as −E_int_ drops down to below 2 kcal/mol, and there is essentially no total intermolecular charge transfer Q.

### 2.3. Less Than Four Substituents

**Figure 3:**
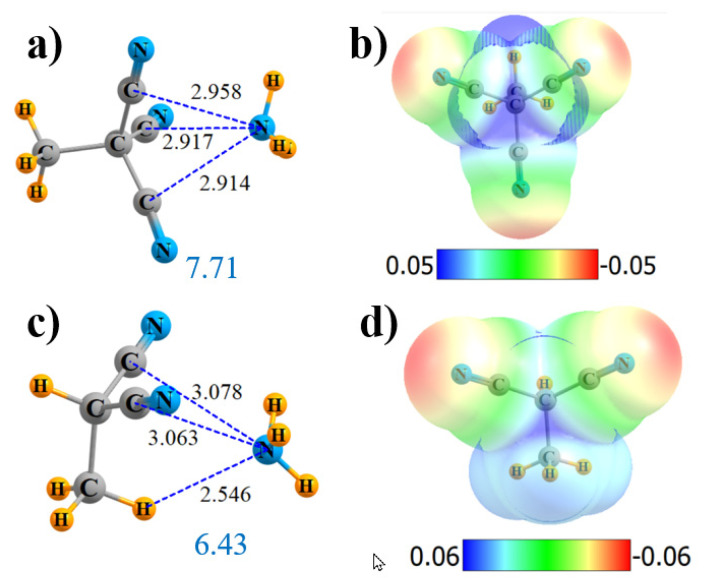
Geometries and monomer MEP of complex of NH_3_ with H_3_C-C(CN)_3_ (**a**,**b**) and H_3_C-CH(CN)_2_ (**c**,**d**).

It is interesting to consider the influence of the three CN groups of the end-on geometry of C_2_(CN)_6_ that do not participate in the bonding to NH_3_, as they lie on the opposite end of the molecule. The three direct interactions remain, as indicated in Figure S3a, with R(N··C) distances of about 2.92 Å in Figure 3a. The MEP retains its maximum along the C-C axis, but it drops in magnitude down to 33.1 kcal/mol, as compared to 46.9 kcal/mol for the same position in the fully substituted C_2_(CN)_6_. There are other reductions as well, including the decrease of the three ρ_BCP_ values, the loss of the N···C bond, the NBO values of E(2), and, also, the total charge transfer Q. Altogether, these effects diminish the interaction energy from 10.3 to 7.7 kcal/mol.

With respect to the side-on configuration, removing one CN group from either C of (CN)_3_C-C(CN)_3_ yielded a weakening of the interaction, as indicated above. One can also remove another pair of groups, leaving (CN)H_2_C-CH_2_(CN). If there is one CN on either C, the maximum in the MEP evaporates, and NH_3_ will not bind in that position but, rather, reorient to engage in a CH··N H-bond. On the other hand, if both CN substituents are left on the same C, their pull on the electrons is sufficient to retain the MEP maximum between the two alkyl C atoms of H_3_C-CH(CN)_2_ (Figure 3d), and the NH_3_ will bind in that position. In addition to the two N··C bonds, the AIM analysis in Figure S3b shows a third bond path, consistent with a CH··N HB, with a length in Figure 3c of only 2.546 Å. Indeed, the densities in Table 1 suggest that this HB may be a bit stronger than the two N··C bonds, a conclusion with which the E(2) quantities concur. 

If all but one of the CN groups is removed, the remaining H_3_C-CH_2_(CN) unit has neither a properly placed V_max_ nor will it engage in a N··C interaction with NH_3_, as witnessed in the entries in Table 1.

### 2.4. Substituted Alkene and Alkyne

**Figure 4:**
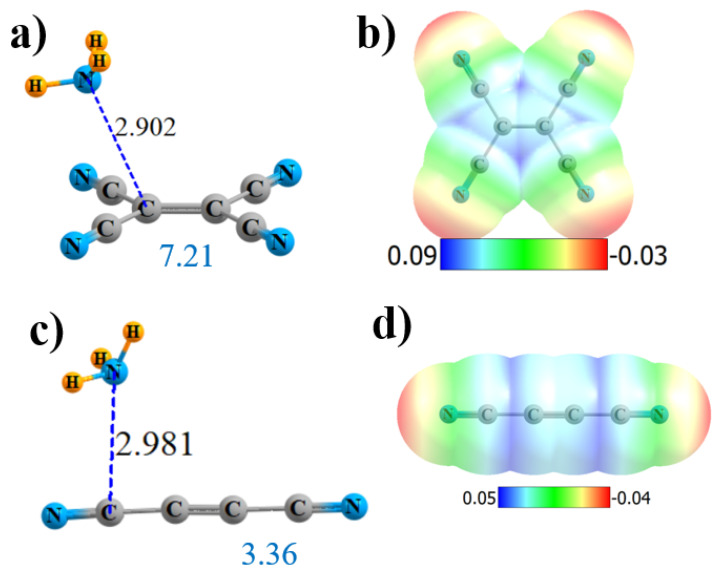
Geometries and monomer MEP of the complex of NH_3_ with (CN)_2_C=C(CN)_2_ (**a**,**b**) and (CN)C≡C(CN) (**c**,**d**).

Another variant might be to place all four CN groups on an alkene, rather than alkane. (CN)_2_C=C(CN)_2_ does have maxima located above the C=C bond, but they are displaced substantially from the bond’s midpoint, as illustrated in Figure 4b. Consequently, NH_3_ is drawn to a position above one of the two C(CN)_2_ groups, with a single bond path to that particular alkene C, with the length 2.902 Å (Figure 4a). The V_max_ at that point is 39.6 kcal/mol, comparable to the alkane derivatives. The bond critical point density is 0.013 au, comparable to the other bonds. NBO suggests not only a strong interaction with the π*(C=C) antibonding orbital but, also, a pair of N··C bonds to the C≡N groups, although the latter are not confirmed by AIM. The total charge transfer is 0.036 e. In sum, the total interaction energy is 7.21 kcal/mol, commensurate with the various quantities in Table 1.

The double-bond can be upgraded to a triple-bond as well. NH_3_ again locates itself off-center in Figure 4c, consistent with the MEP of the Lewis acid in Figure 4d. AIM identifies a single-bond path that leads to a C_N_ atom, with R = 2.981 Å and with ρ_BCP_ = 0.010 au. NBO shows two π* sinks of the charge transferring from the N lone pair: some go into the C≡C antibond and some into C≡N. The interaction energy is about half that for the alkene, in keeping with the lowered V_max_, ρ_BCP_, E(2), and Q.

### 2.5. Cyclic Lewis Acids

**Figure 5:**
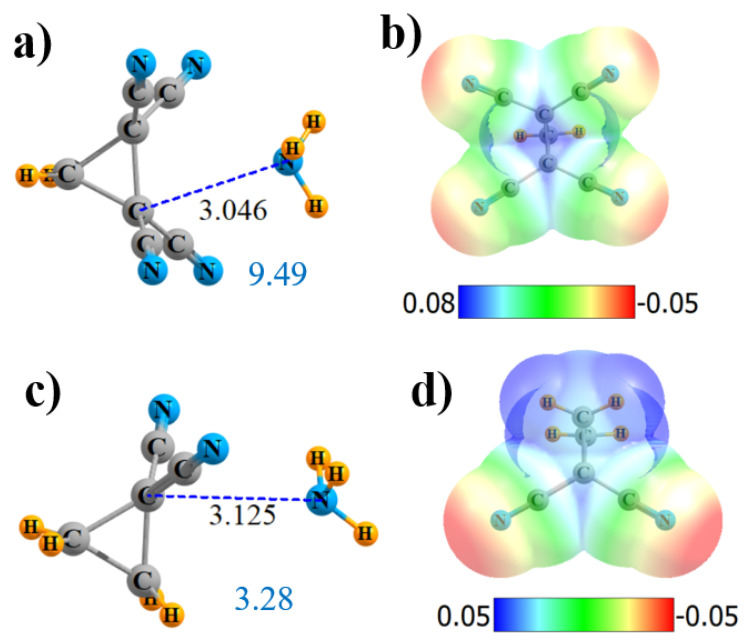
Geometries and monomer MEP of the complex of NH_3_ with cyclic Lewis acids CH_2_{C(CN)_2_}_2_ (**a**,**b**) and CH_2_CH_2_C(CN)_2_ (**c**,**d**).

Some of the more interesting observations of these sorts of CN interactions were derived from cyclopropane derivatives. Hence, molecules of the sort illustrated in Figure 5 were considered. Although the MEP in Figure 5b is rather symmetric, and NH_3_ accordingly positioned, AIM provides only a single-bond path, which connects with none of the CN groups but, rather, to one of the alkane C atoms. The NBO picture is rather different, indicating not only bonds to both alkane C atoms but to all four CN groups as well. Each of the former involve a σ*(CC)-antibonding orbital of the cycloalkane, as exhibited in Figure S2d. The σ-hole depth in this cyclical molecule is 36.7 kcal/mol, slightly shallower than in its noncyclical (CN)_2_HC-CH(CN)_2_ parallel. Likewise for the other parameters: fewer AIM bond paths, smaller E(2), and lower Q, all conflate to a weaker binding of 9.49 kcal/mol vs. 11.80 kcal/mol for the noncyclical analogue.

Just as in the former noncyclical systems, removing both of the CN groups from one of the C atoms also weakens the σ-hole. Like the tetrasubstituted derivative, AIM provides only a single-bond path, again to one of the alkane C atoms, which is 3.125-Å removed, as indicated in Figure 5c. NBO maintains both of the bonds to the alkane C atoms, as well as to both C_N_. The total charge transfer is reduced nearly in half by the removal of two CN groups, as is the interaction energy. 

The replacement of the four CN groups of cyclic CH_2_{C(CN)_2_}_2_ by F atoms again weakens all aspects of the interaction: V_max_, ρ_BCP_, E(2), and Q. However, it does offer a new wrinkle in terms of the AIM bond path. Instead of four N··F bond paths, as was observed for the noncyclic system, Figure S4c shows that the single bond path extends in the cyclic system from N to the midpoint of the C-C axis (although it veers off to one of the C atoms at the very last minute). NBO suggests an alternative perspective of transfers to the two C-C-antibonding orbitals of the remainder of the ring. All aspects of the interaction are reduced relative to the tetracyano analog, leaving the interaction energy at 3.28 kcal/mol. 

**Figure 6:**
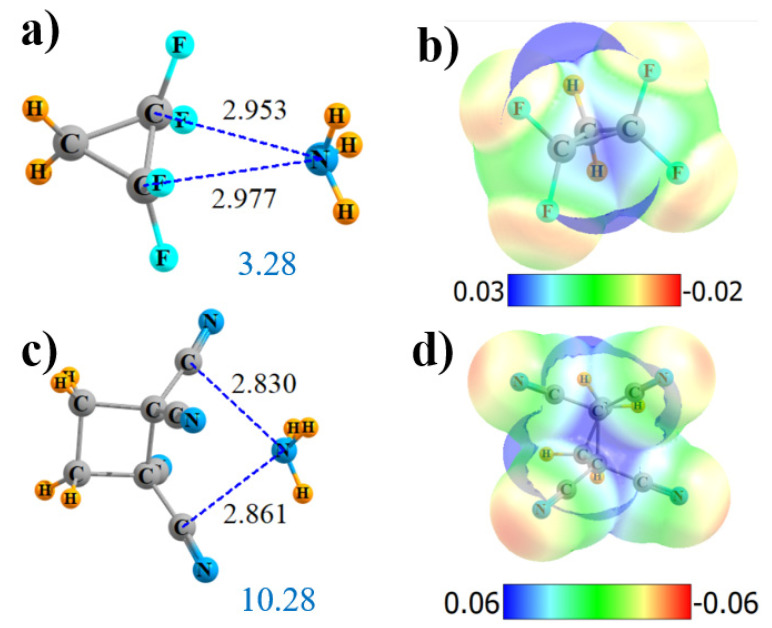
Geometries and monomer MEP of the complex of NH_3_ with cyclic Lewis acids CH_2_{CF_2_}_2_ (**a**,**b**) and CH_2_CH_2_{C(CN)_2_}_2_ (**c**,**d**).

Another variation that was tested enlarged the ring to cyclobutane. This enlargement drastically altered the AIM picture of bonding, bypassing the ring C atoms entirely, and suggesting instead a pair of N··C_N_ interactions, as pictured in Figure S4d. These bonds are 2.8 Å in length, as may be seen in Figure 6c. In this case, NBO confirms the AIM conclusions with a pair of bonds to the π*(CN) antibonds. The MEP maximum in Figure 6d lies roughly at the center of one of the C-C bonds. The extension from three to four C atoms in the ring slightly strengthens the overall interaction energy from 9.49 to 10.28 kcal/mol, despite a small reduction in V_max_. The slack is taken up by a larger ρ_BCP_, E(2), and total charge transfer Q.

### 2.6. Monomer Perturbations

One of the more interesting facets of intermolecular interactions is their effect upon the properties of the individual subunits. The geometric perturbations introduced into the Lewis acids are reported in Table 2 in terms of the changes in the various bond lengths. The C-C designation refers to the bond between the two alkyl C atoms (or alkene or alkyne) that are not part of the C≡N substituents. The bond between this alkyl C and the cyano C is labeled as C-C_N_, while C_N_≡N refers to the bond within the cyano group. The values displayed in Table 2 represent an average of all the cyano groups that are involved in the interaction with NH_3_—for example, all four in Figure 1a and three in Figure 1b.

From the first column of Table 2, it is clear that most of the interactions lead to a contraction of the alkyl C-C bond. Most of these bond shortenings are fairly small, with the principal exception of the end-on arrangement with (CN)_3_C-C(CN)_3_, where the bond contracts by 0.045 Å. There are two exceptions to this rule, in that the bond elongates for the end-on arrangement of H_3_C-C(CN)_3_ and an even smaller stretch for CH_3_-CH(CN)_2_. The C-C_N_ bond connecting the cyano group to the alkyl chain undergoes only minor modifications and of variable signs. The principal exception is a 0.007-Å stretch for CH_3_-CH(CN)_2_. Due to the charge transfer into many of the π*(C≡N)-antibonding orbitals, the C_N_≡N bonds stretch but surprisingly little, changing by less than 1 mÅ.

Along with the changes in C≡N bond lengths, one can also expect certain perturbations in their internal stretching frequencies. Since there are several such groups on each Lewis acid molecule, the normal modes representing their stretches will be highly coupled with one another. The C≡N stretching band will thus encompass n individual modes, where n is equal to the number of such groups. The changes induced by the complexation of NH_3_ with each of the Lewis acids on the minimum, maximum, and mid-range C≡N stretching frequencies are presented in Table 3. In most cases, there is a broadening of the full band with the minimum dropping by several wavenumbers and the maximum increasing. In terms of the center of the band, there is a blue shift of several cm^−1^, with two exceptions.

Another type of perturbation induced by complexation relates to NMR chemical shielding. It is widely understood, for example, that the formation of a H-bond reduces the shielding around the bridging proton and shifts its signal downfield. The changes in the chemical shielding of the various atoms resulting from the complexation with NH_3_ are displayed in Table 4. The shielding of the C atoms of the C≡N groups are diminished by a fairly uniform amount of about 2 to 3 ppm, regardless of the strength of the interaction. Indeed, there is no clear relation between these two quantities. For example, the largest deshielding of 2.90 ppm occurs for the cyclic CH_2_CH_2_C(CN)_2_ system that engages in one of the weakest interactions.

The N atom of the C≡N groups undergoes a substantial additional shielding of as much as 12 ppm upon complexation with NH_3_. There is a general relationship of higher shielding for stronger interactions, but this relationship is a very weak one; the correlation coefficient R^2^ is only 0.3. However, NBO charge transfers offer a better parameter to compare with the N shielding. For example, the largest value of E(2) for transfer into a π*(C≡N)-antibonding orbital is 2.3 kcal/mol for the end-on (CN)_3_C-C(CN)_3_ complex, and it is in this structure that N undergoes its maximum shielding increase of 12.2 ppm. The smallest shielding increase of 4.0 ppm occurs in H_3_C-CH(CN)_2_, and it this same system that has the smallest E(2) of 0.5 kcal/mol.

Like the cyano C_N_ atom, the connecting alkyl C suffers a loss of shielding in all cases. However, unlike C_N_, the deshielding of C is highly variable from as little as 0.4 ppm to a maximum of 9.7 ppm. The NBO analyses again offer some insight into the shifts of this connecting C. The connecting C-C (or C=C) antibond acts as charge acceptor in the end-on (CN)_3_C-C(CN)_3_ complex, as well as the one involving the alkene, and it is for those complexes that the C atom suffers its largest deshielding of more than 7 ppm. (On the other hand, the shielding drop is only 2.6 ppm for the alkyne, even though the C≡C antibond is involved in a NBO transfer.) This same concept extends to the cyclic complexes as well. Both tetra- and di-substituted cycloalkanes see a sizable deshielding of the ring C atoms, and, in both cases, there is transfer into the C-C antibond. Based upon the similarities between the behavior of the noncyclic and cyclic systems, the strain introduced by the formation of a small ring does not appear to produce any dramatic effects upon the changes in the shielding shift. On the other hand, the cyclopropyl derivatives do have a somewhat larger absolute shielding on their ring C atoms than is the case in the noncyclic systems.

## 3. Discussion

Despite a number of basic similarities, the complexes are held together by a surprisingly wide array of noncovalent bonds. There are, first of all, AIM bond paths connecting the base N atom to the C atoms of the C≡N groups. The NBO analysis indicates that charge is being transferred into the π*(CN)-antibonding orbitals, so these interactions would fit into the tetrel bond classification, more in the nature of a π-hole than a σ-hole. There are also bond paths that lead to the bridging alkyl C atoms in a somewhat different sort of tetrel bond, as it is a σ*-antibonding orbital that acts as charge receptor, both σ*(CC) and σ*(CH). In the case of alkene and alkyne acids, the π*(CC)-antibonding orbitals of the bridging atoms act in this same capacity. The switch from C≡N to F substituents provides bond paths from ammonia to these F atoms. As NBO designates the σ*(C-F)-antibonding orbitals as the destination of the charge, these interactions might be described as a halogen bond, albeit an unusual and highly bent one.

Whereas most noncovalent bonds can generally be clearly subdivided into either σ or π-hole types, these systems are more complicated. The site of the maximum in the MEP into which the base nestles is not the product of any one particular covalent bond polar flattening or electron deficiency, nor is it associated with any single substituent. It might be better thought of as the coalescence of several π-holes, one from each of the two to four C≡N groups, plus any positive MEP that might be associated with the connecting alkyl C-C, or C=C or C≡C, depending on the particular molecule. An exception that proves the rule comes from the alkyne (CN)C≡C(CN) case. The disposition of the two C≡N groups on opposite ends of the molecule prevents them from pooling their two π-holes, and so the MEP maximum is much shallower than in the other cases, only 22 kcal/mol. The resulting interaction energy is also weak, only 3.4 kcal/mol. In any case, regardless of its origin, the intensity of this positive MEP region is closely connected with the interaction energy with NH_3_. 

Given that F is comparable in terms of the electron-withdrawing capacity to C≡N, with nearly equal inductive Hammet sigma constants [67] (although the two differ in terms of σ_p_), there is a question as to why the cyano-substituted Lewis acids form so much more intense MEP maxima and stronger interactions with NH_3_ than do their F counterparts. Taking the simple MeF and MeCN as examples of simpler model acids, the σ-hole generated opposite the F atom along the F-C extension in MeF is equal to 19.7 kcal/mol, only slightly less than the equivalent σ-hole of 23.1 kcal/mol for MeCN. This similarity extends also to the interaction energies of these two acids with NH_3_, barely different at 2.16 vs. 2.31 kcal/mol, respectively.

However, there is more to the ability of the substituent to adjust the MEP maximum than its pure electron-withdrawing power. One must also consider the positional aspects of the MEP, particularly as the MEP maxima in the Lewis acids discussed above fall far from a σ-hole position along any particular bond. The maximum of the MEP occurs for F_2_HC-CHF_2_ in Figure 2 at a θ(CF-p) angle of 67°, where p represents the position of the MEP maximum, and p is located 1.92 Å from F. The comparable θ(CC··p) angle and R(C··p) are 66° and 2.20 Å for (CN)_2_HC-CH(CN)_2_. The MEP of the simple MeF monomer at this same point relative to the C-F bond is +9.3 kcal/mol, as compared to more than twice that amount, 20.9 kcal/mol, for MeCN. Therefore, one can attribute the much larger MEP maxima for the cyano-substituted systems to the more positive MEP that lie well off the C-C axis. It is this more intense MEP that is, in large part, responsible for the stronger binding of C≡N vs. F substituted systems.

**Figure 7:**
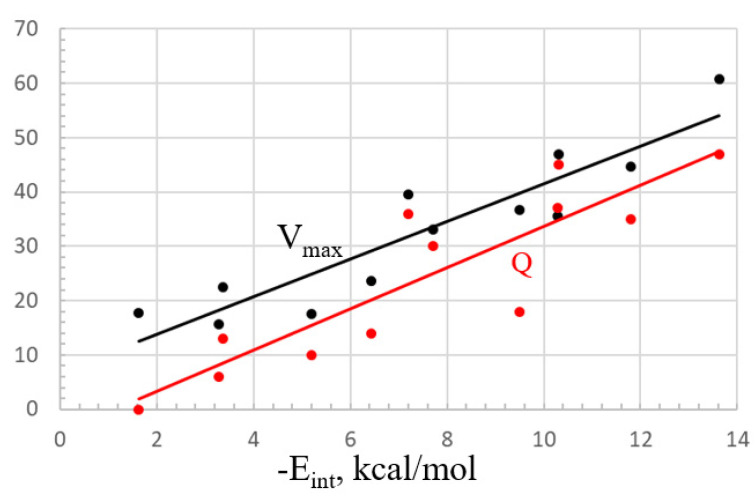
Linear fitting of the interaction energy to the MEP maximum (kcal/mol, black) and total charge transfer Q (red, me).

In fact, among the various properties, it would appear that the interaction energy is most closely aligned with the value of the MEP maximum. The best-fit linear relationship between these two quantities is illustrated by the black curve in Figure 7. The fit is good but not perfect, with a correlation coefficient of R^2^ = 0.85. There is also a solid relationship between E_int_ and the total charge transfer Q, but this is less linear, with an R^2^ of only 0.78, illustrated by the red line in Figure 7.

A primary distinguishing feature of these molecules is their ability to “pool” their π-hole-positive C≡N regions together. For example, the simple MeC≡N does not have a pure π-hole above its C≡N bond but, rather, a vaguely diffuse positive region that sits above the C-C bond rather than C≡N. Replacing the methyl group by an electron-withdrawing halogen atom leaves this positive area in place [62], again displaced away from C≡N. When two of these C≡N units are connected through an intervening C≡C triple-bond, as in (CN)C≡C(CN), and are separated from one another, the most positive region remains in this same position—in this case, above the connecting C-C bond, as seen in Figure 4d. This π-hole is shallow, with V_max_ = 22 kcal/mol. It is only when these C≡N groups are situated in closer proximity to one another that they can pool their positive regions. The (CN)_2_C=C(CN)_2_ alkene is an example, where Figure 4b shows a positive region accumulating between each pair of cyano groups, closer to the C atoms, and V_max_ nearly doubles to 40 kcal/mol. The cumulative effect reaches its culmination when there are three or four adjoining cyano groups, and V_max_ rises up above 45 and even above 60 kcal/mol.

**Figure 8:**
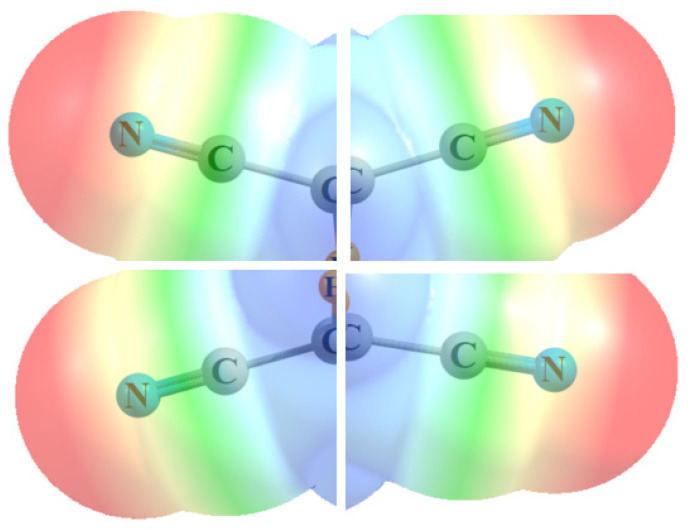
Partial MEP of MeC≡N in four different orientations, pieced together so as to compare with the MEP of (CN)_2_HC-CH(CN)_2_ in Figure 2b.

The way in which the various C-C≡N units are able to bring their individual MEPs together so as to create the centralized positive regions in the figures might best be visualized in Figure 8. Each of the four segments represents the MEP calculated for the simple MeC≡N molecule. Simply by bringing these MEP segments together in the proper alignment, as would occur in a tetra-substituted molecule like (CN)_2_HC-CH(CN)_2_, with no other manipulation or perturbation, one is able to mimic the principal feature of the actual MEP of the entire molecule, as in Figure 1b and Figure 2b, or Figure 5b.

Aside from electrostatics, there are clearly other attractive forces in these complexes. The NBO and Q parameters are contained within the induction contribution, for example. These various terms can be quantified in an energetic sense via SAPT decomposition of the total interaction energy. The attractive electrostatic (ES), induction (IND), and dispersion (DISP) terms are listed in Table 5 for the selected complexes. The configurations span a wide range, from four to two interacting C≡N groups, and both cyclic and noncyclic molecules, containing F, as well as C≡N, substituents. Of course, the various terms diminish along with the overall strength of each interaction. The ES contribution amounts to more than 22 kcal/mol for the most strongly bound side-on complex with (CN)_3_C-C(CN)_3_. The induction and dispersion terms are also sizable, at 16.2 and 10.4 kcal/mol, respectively. These quantities are reduced to 7.4, 3.9, and 4.7 kcal/mol, respectively, for the cyclic CH_2_CH_2_C(CN)_2_ with only two C≡N contacts. However, what is perhaps most striking is the consistency of their relative contributions. As indicated in the last three columns of Table 5, the ES accounts to close to half of the total attractive force, while the IND makes up about 30%, followed by DISP at just over 20%. The only significant deviation occurs for cyclic CH_2_CH_2_C(CN)_2_, with n = 2, where there is a boost of the dispersion to 30% and a corresponding drop in the induction.

However, the situation changes when C≡N substituents are replaced by F. As is evident in the last two rows of Table 5, this switch drops the ES proportion of the attractive energy down below 40%, with a compensating rise in induction energy of 37%. This disproportionate decrease in ES is consistent with the much lower V_max_ for these F species, below 18 kcal/mol, which underscores the distinction between C≡N and F substituents in the binding of a base.

As noted above, there is some erratic character as to the precise connecting points of the AIM bond paths connecting NH_3_ to the Lewis acid. This finding brings to mind earlier works that have brought into question the ability of AIM paths to unambiguously identify bonding interactions [68,69,70,71].

There is always the question as to the sensitivity of the computed data to the particular basis set chosen. For this reason, one particular complex, pairing NH_3_ with H_3_C-C(CN)_3_, was taken as a test system, and the results were recomputed with a larger triple-valence set. The relevant data are summarized in Table 6 and show stability to the basis set choice. The interaction energy, in particular, shows little change upon adding the extra set of basis functions. Upgrading the electron correlation method to CCSD(T) produces a small reduction in the interaction energy, but part of this lowering arises from the somewhat less flexible 6-311+G* basis set. In terms of NMR data, the chemical shifts were recomputed with the aug-cc-pCVTZ basis set (and aug-cc-pVTZ for H), which ought to provide a better account of the inner shell orbitals. The shifts displayed in parentheses in Table 6 verify little perturbations from basis set choice. As a further test of the stability of the AIM bond paths, the analysis of the aug-cc-pVTZ density duplicates the same paths with the double-zeta set and with nearly precisely equal critical point densities. This similarity applies not only to H_3_C-C(CN)_3_ but, also, to the complexes pictured in Figure 3c, Figure 4a,c and Figure 5c.

The earlier work by the Mooibroek and Frontera groups [23,63,64,65,66] focused attention on the CN-substituted cyclopropane system, as well as larger cyclic chains, as an effective Lewis acid. The calculations described above demonstrate that the ability to engage in bonding of this type is not limited to cyclic C chains, with their accompanying strain, but is rather a general feature of the placement of one or more C≡N substituents on adjacent C atoms, even if they are multiple-bonded to one another. Indeed, the relaxation of the cyclic constraint enhances the interaction energy to a certain degree.

As a final point, there is experimental verification of the ability of the π-cloud of the C≡N bond of acetonitrile to engage in a tetrel bond with nucleophiles, derived from an analysis of numerous crystal structures [61]. Two such examples from that work are displayed in Figure S5.

## 4. Conclusions

The aggregation of several C≡N groups on a single molecule, situated in such a way that they can pool the positive regions of their individual electrostatic potentials, results in rather strong interactions with a nucleophile. In the strongest such case, four of the six cyano groups on (CN)_3_C-C(CN)_3_ bind to NH_3_ with a total interaction energy of 13.6 kcal/mol. Even though F is comparable to C≡N in terms of electron-withdrawing power, it is unable to develop the same level of positive potential off of the C-F axis, so engages in much weaker interactions with a base. The precise nature of the bonding is variable, although tetrel bonding is a common theme. This tetrel bonding can involve either the cyano C atom or the alkyl C atoms of the spacer C-C bond. There is some variability also in terms of the orbital that accepts a charge from the N lone pair. The π*(CN) orbitals in the cyano groups are the most common acceptor, but various σ*-antibonding orbitals are also used. The latter include the C-C_N_ bond to the cyano group, C-H, and C-F, as well as the C-C, C=C, and C≡C-antibonding orbitals of the alkane, alkene, and alkyne, respectively. Rearranging the substituted alkane into a cyclic molecule has a mild moderating effect on the interaction.

## 5. Computational Methods

Calculations were carried out with the M06-2X DFT functional applied to the aug-cc-pVTZ basis set, within the framework of the Gaussion-09 set of codes [70]. Interaction energies were derived as the difference in energy between the complex and the sum of the two monomers in the geometry they adopt within the dimer. This quantity was then corrected for basis set superposition error through application of the counterpoise [71] protocol. The molecular electrostatic potential (MEP) was analyzed via the Multiwfn program [72], so as to identify the positions and values of the MEP at its maximum on an isodensity surface with ρ = 0.001 au. The topology of the electron density was analyzed for bond paths via the AIMALL program [73]. Natural bond orbital (NBO) formalism extracted the atomic charges and energetic measures of the charge transfer between orbitals [74,75]. NMR chemical shielding was evaluated under the GIAO approximation [76,77,78]. Interaction energies were decomposed into individual components via the symmetry-adapted perturbation theory (SAPT) [79,80,81,82], as implemented in the Molpro program [83].

## Figures and Tables

**Table 1:** Interaction energy, maximum of monomer molecular electrostatic potential (MEP), bond critical point density, natural bond orbital (NBO) charge transfer energy, and total charge transferred in complexes with NH_3_.

Lewis Acid	N ^a^	−E_int_, kcal/mol	V_max_, kcal/mol	ρ_BCP_ × 10^4^, au ^b^	E(2), kcal/mol ^c^	Q ^d^, e
noncyclic						
(CN)_3_C-C(CN)_3_ side	4	13.63	60.7	4 × C_N_ 130	4 × C≡N 1.4 2 × C-C_N_ 0.7	0.047
(CN)_3_C-C(CN)_3_ end	3	10.30	46.9	3 × C_N_ 135 C 131	3 × C≡N 2.3 C-C 0.9	0.045
(CN)_2_HC-CH(CN)_2_	4	11.80	44.7	2 × C_N_ 118 C 111	4 × C≡N 1.4 2 × CH 0.6	0.035
F_2_HC-CHF_2_	4	1.63	17.7	4 × F 90	4 × CF 0.2 2 × CH 0.2	0.000
H_3_C-C(CN)_3_ end	3	7.71	33.1	3 × C_N_ 120	3 × C≡N 1.8 C-C 0.5	0.030
(CN)H_2_C-CH_2_(CN)	2	CH--N	-			
H_3_C-CH(CN)_2_	2	6.43	23.7	CH··N 112 2 × C_N_ 86	CH··N 2.7 2 C≡N 0.5	0.014
H_3_C-CH_2_(CN)	1	CH--N	-			
(CN)_2_C=C(CN)_2_	2	7.21	39.6	C 133	C=C 2.2 2 × C≡N 0.9	0.036
(CN)C≡C(CN)	1	3.36	22.4	C_N_ 100	C≡C 0.7 C≡N 0.9	0.013
cyclic						
CH_2_{C(CN)_2_}_2_	4	9.49	36.7	C 115	2 × CC 0.5 4 × C≡N 0.5	0.018
CH_2_CH_2_C(CN)_2_	2	5.19	17.5	C 89	CC 0.4 2 × C≡N 0.5	0.010
CH_2_{CF_2_}_2_	4	3.28	15.6	C 126	2 × CC 1.0	0.006
CH_2_CH_2_{C(CN)_2_}_2_	4	10.28	35.6	2 × C_N_ 132	2 × CC 0.6 4 × C≡N 1.1	0.037

^a^ Number of substituents (CN or F) close to NH_3_. ^b^ Bond path involving NH_3_ N and indicated atom on Lewis acid. ^c^ Shift from N lone pair to indicate antibonding orbitals of Lewis acid. ^d^ Sum of natural atomic charges on the NH_3_ subunit.

**Table 2:** Changes in internal bond lengths ^a^ (Å) caused by complexation with NH_3_.

Lewis Acid	C-C	C-C_N_	C_N_≡N
noncyclic			
(CN)_3_C-C(CN)_3_ side	−0.0098	0.0002	0.0001
(CN)_3_C-C(CN)_3_ end	−0.0451	−0.0005	0.0001
(CN)_2_HC-CH(CN)_2_	−0.0075	−0.0004	0.0002
H_3_C-C(CN)_3_ end	0.0011	−0.0010	0.0003
H_3_C-CH(CN)_2_	0.0005	0.0074	−0.0003
(CN)_2_C=C(CN)_2_	−0.0024	0.0000	−0.0002
(CN)C≡C(CN)	−0.0002	0.0007	0.0000
cyclic			
CH_2_{C(CN)_2_}_2_	−0.0123	−0.0006	0.0002
CH_2_CH_2_C(CN)_2_	−0.0072	−0.0010	0.0003
CH_2_CH_2_{C(CN)_2_}_2_	−0.0019	−0.0024	0.0003

^a^ Averages when more than one pertinent bond interacts with NH_3_.

**Table 3:** Changes in C≡N vibrational frequencies (cm^−1^) caused by complexation with NH_3_.

Lewis Acid	Min	Max	Center
(CN)_3_C-C(CN)_3_ side	0.4	9.2	4.8
(CN)_3_C-C(CN)_3_ end	3.0	5.0	4.0
(CN)_2_HC-CH(CN)_2_	−5.2	11.0	2.9
H_3_C-C(CN)_3_ end	−6.7	3.5	−1.6
H_3_C-CH(CN)_2_	−4.3	6.5	1.1
(CN)_2_C=C(CN)_2_	−0.7	−2.2	−1.4
(CN)C≡C(CN)	7.9	2.3	5.1

**Table 4:** Changes in NMR chemical shielding ^a^ (ppm) caused by complexation with NH_3_.

Lewis Acid	C	C_N_	N
noncyclic			
(CN)_3_C-C(CN)_3_ side	−1.72	−1.97	10.66
(CN)_3_C-C(CN)_3_ end	−9.67	−1.83	12.19
(CN)_2_HC-CH(CN)_2_	−1.67	−2.10	9.11
H_3_C-C(CN)_3_ end	−5.20	−2.88	6.33
H_3_C-CH(CN)_2_	−0.41	−2.86	4.04
(CN)_2_C=C(CN)_2_	−7.42	−1.82	11.05
(CN)C≡C(CN)	−2.63	−1.99	5.90
cyclic			
CH_2_{C(CN)_2_}_2_	−5.00	−2.76	6.03
CH_2_CH_2_C(CN)_2_	−5.95	−2.90	5.14
CH_2_CH_2_{C(CN)_2_}_2_	−0.81	−2.82	5.66

^a^ Averages when there is more than one pertinent atom.

**Table 5:** Symmetry-adapted perturbation theory (SAPT) partitioning of the interaction energies in complexes with NH_3_, along with their percentage contribution to the total of all three attractive terms. ES: electrostatic, IND: induction, and DISP: dispersion.

Lewis Acid		kcal/mol			Percentage		
	n	ES	IND	DISP	ES	IND	DISP
(CN)_3_C-C(CN)_3_ side	4	22.37	16.17	10.37	45.7	33.1	21.2
(CN)_2_HC-CH(CN)_2_	4	18.61	12.83	9.01	46.0	31.7	22.3
cyclic CH_2_{C(CN)_2_}_2_	4	13.49	8.41	6.89	46.9	29.2	23.9
(CN)_3_C-C(CN)_3_ end	3	17.65	12.69	7.54	46.6	33.5	19.9
cyclic CH_2_CH_2_C(CN)_2_	2	7.40	3.86	4.71	46.3	24.2	29.5
F_2_HC-CHF_2_	4	4.98	5.45	4.25	33.9	37.1	29.0
cyclic CH_2_{CF_2_}_2_	4	7.53	7.17	4.60	39.0	37.1	23.8

**Table 6:** Quantities calculated by two different basis sets for complex of H_3_C-C(CN)_3_ with NH_3_.

	aug-cc-pVDZ	aug-cc-pVTZ
Δν(C≡N), cm^−1^		
min	−6.7	−5.2
max	+3.5	+0.9
center	−1.6	−2.1
Δσ, ppm		
C	−5.2	−7.9 (−6.3) ^a^
C_N_	−2.9	−2.4 (−2.7) ^a^
N	6.3	7.1 (7.2) ^a^
−E_int_, kcal/mol	7.71	7.77 (5.60) ^b^

^a^ Aug-cc-pCVTZ. ^b^ CCSD(T)/6-311+G*.

## Supplementary Materials

The following are available online, Figure S1: AIM bond paths in complexes of NH_3_ with (CN)_3_C-C(CN)_3_, Figure S2: Overlap of NH_3_ N lone pair orbital, Figure S3: AIM bond paths in complexes of NH_3_, Figure S4: AIM bond paths in complexes of NH_3_ with cyclic.
