# Supplementary material for: Versatility of the Cyano Group in Intermolecular Interactions
Source: Molecules. 2020 Sep 30;25(19):4495. doi: 10.3390/molecules25194495 (PMC7582283; doi:10.3390/molecules25194495)
Supplement: Supplementary file 1 [file molecules-25-04495-s001.pdf]

SUPPLEMENTARY INFORMATION

Versatility of the Cyano Group in Intermolecular Interactions

Steve Scheiner*
Department of Chemistry and Biochemistry
Utah State University
Logan, UT 84322-0300

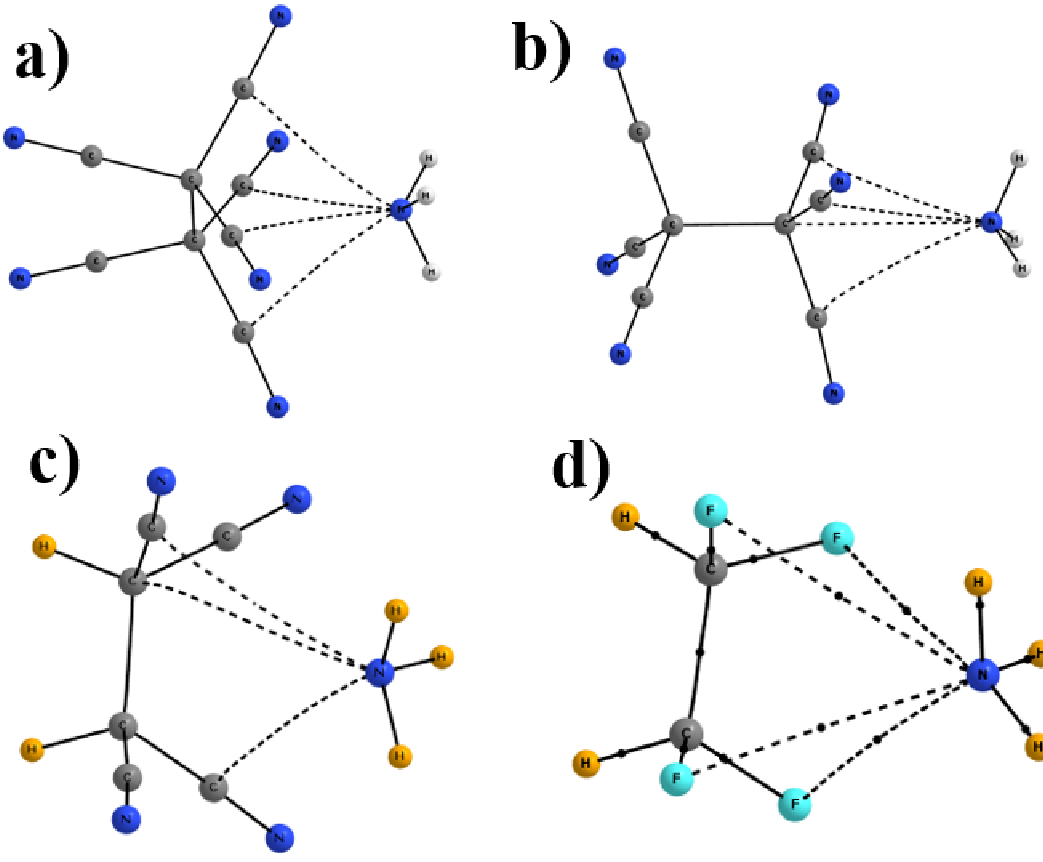

Fig S1. AIM bond paths in complexes of NH_3 with $(CN)_3C\text{-}C(CN)_3$ in a) side-on and b) end-on complexes, c) $(CN)_2HC\text{-}CH(CN)_2$ and d) $F_2HC\text{-}CHF_2$

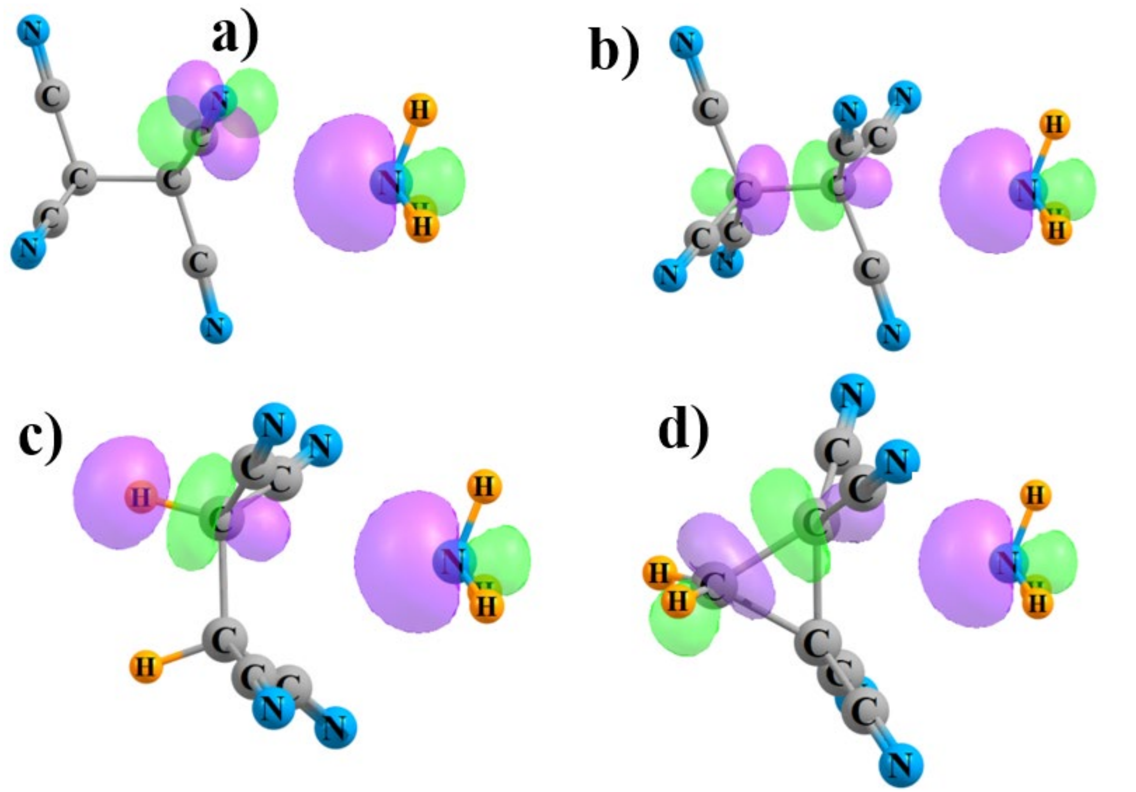

Fig S2. Overlap of NH_3 N lone pair orbital with a) π*(C≡N), b) σ*(C-C), c) σ*(H-C), and d) σ*(C-C) orbital of cyclopropane derivative.

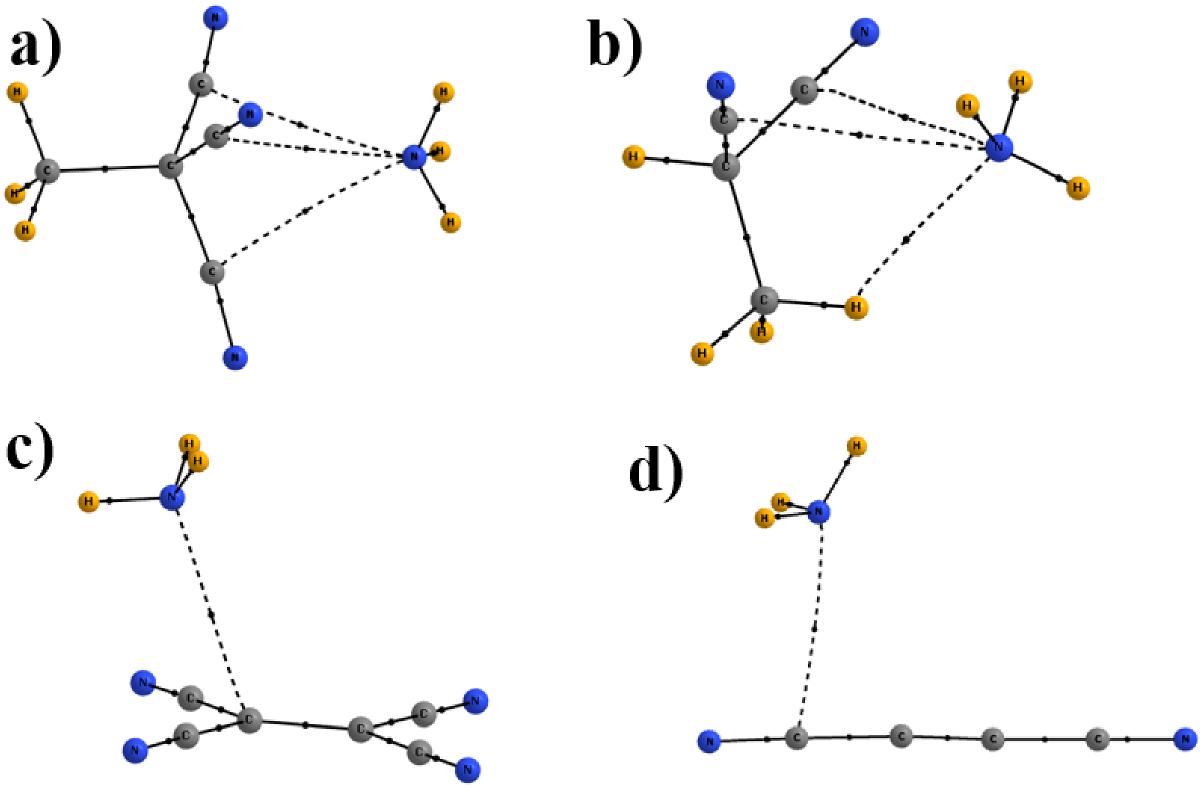

Fig S3. AIM bond paths in complexes of NH_3 with a) $H_3C\text{-}C(CN)_3$, b) $H_3C\text{-}CH(CN)_2$, c) $(CN)_2C{=}C(CN)_2$ and d) $(CN)C{\equiv}C(CN)$.

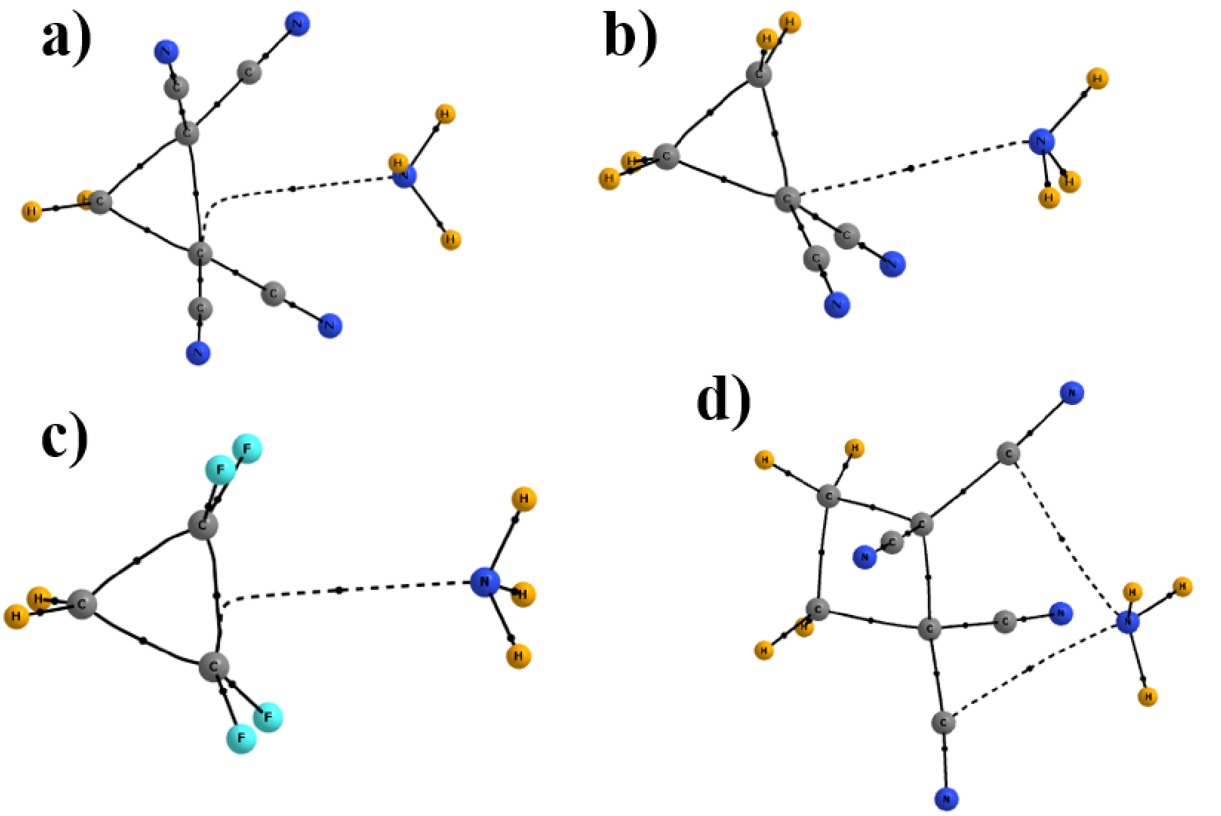

Fig S4. AIM bond paths in complexes of NH_3 with cyclic a) $CH_2\{C(CN)_2\}_2$, b) $CH_2CH_2C(CN)_2$, c) $CH_2\{CF_2\}_2$ and d) $CH_2CH_2\{C(CN)_2\}_2$.

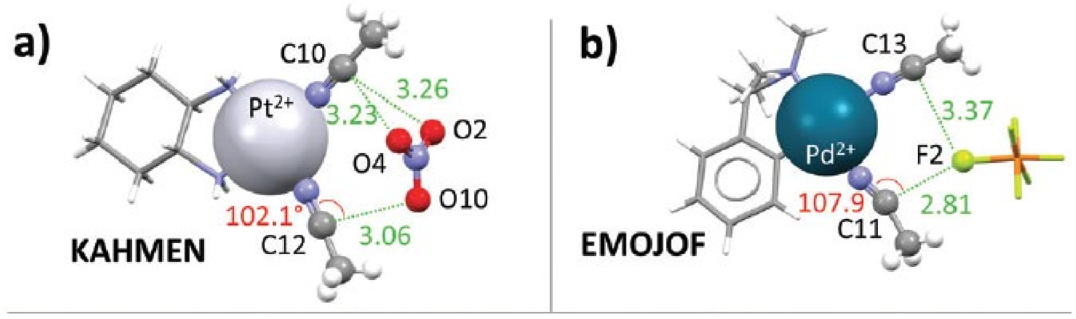

Fig S5. Examples of crystal structures containing short contacts between C≡N groups and potential electron donors. Reproduced from Ref. 61 with permission from The Royal Society of Chemistry.
